# Induction of proteasome expression in skeletal muscle is attenuated by inhibitors of NF-*κ*B activation

**DOI:** 10.1038/sj.bjc.6602165

**Published:** 2004-10-12

**Authors:** S M Wyke, S T Russell, M J Tisdale

**Affiliations:** 1 Pharmaceutical Sciences Research Institute, Aston University, Birmingham B4 7ET, UK

**Keywords:** cancer cachexia, muscle wasting, NF-*κ*B, proteasome expression

## Abstract

The potential for inhibitors of nuclear factor-*κ*B (NF-*κ*B) activation to act as inhibitors of muscle protein degradation in cancer cachexia has been evaluated both *in vitro* and *in vivo*. Activation of NF-*κ*B is important in the induction of proteasome expression and protein degradation by the tumour factor, proteolysis-inducing factor (PIF), since the cell permeable NF-*κ*B inhibitor SN50 (18 *μ*M) attenuated the expression of 20S proteasome *α*-subunits, two subunits of the 19S regulator MSS1 and p42, and the ubiquitin-conjugating enzyme, E2_14k_, as well as the decrease in myosin expression in murine myotubes. To assess the potential therapeutic benefit of NF-*κ*B inhibitors on muscle atrophy in cancer cachexia, two potential inhibitors were employed; curcumin (50 *μ*M) and resveratrol (30 *μ*M). Both agents completely attenuated total protein degradation in murine myotubes at all concentrations of PIF, and attenuated the PIF-induced increase in expression of the ubiquitin–proteasome proteolytic pathway, as determined by the ‘chymotrypsin-like’ enzyme activity, proteasome subunits and E2_14k_. However, curcumin (150 and 300 mg kg^−1^) was ineffective in preventing weight loss and muscle protein degradation in mice bearing the MAC16 tumour, whereas resveratrol (1 mg kg^−1^) significantly attenuated weight loss and protein degradation in skeletal muscle, and produced a significant reduction in NF-*κ*B DNA-binding activity. The inactivity of curcumin was probably due to a low bioavailability. These results suggest that agents which inhibit nuclear translocation of NF-*κ*B may prove useful for the treatment of muscle wasting in cancer cachexia.

Loss of skeletal muscle mass in cancer patients has a negative influence on prognosis, resulting in asthenia, immobility and early death. While lysosomal and calcium-activated proteases play some role in the breakdown of myofibrillar proteins, the ubiquitin–proteasome proteolytic pathway is considered to be the predominant system in both experimental models of cancer cachexia (Attaix *et al*, 1999) and in cancer patients (Bossola *et al*, 2003). Proteins to be degraded are marked by the covalent attachment of a polyubiquitin chain, which is recognised by the 19S subunits of the 26S proteasome, a multisubunit cylindrical structure composed of *α* and *β* units, with the proteolytic enzymes situated on the inner surface of the cylinder on the *β*-subunits. Expression of the enzymes of ubiquitin conjugation and proteasome subunits are upregulated in cancer cachexia (Lorite *et al*, 1998). Knowledge of the mechanism for the regulation of proteasome expression in cancer cachexia should facilitate the design of effective inhibitors.

Muscle mass in cancer cachexia is strongly regulated by a tumour produced sulphated glycoprotein, proteolysis-inducing factor (PIF), which inhibits protein synthesis and stimulates protein degradation (Lorite *et al*, 1997). The effect on protein degradation is due to an increased gene and protein expression of the key components of the ubiquitin–proteasome proteolytic pathway (Lorite *et al*, 2001). In murine myotubes, induction of proteasome expression by PIF is associated with an increased DNA-binding activity of the transcription factor NF-*κ*B, concomitant with a transient decrease in cytosolic levels of the NF-*κ*B inhibitor protein, I-*κ*B*α* (Whitehouse and Tisdale, 2003). Degradation of I-*κ*B*α* is initiated by phosphorylation of two serine residues (32 and 36) in the N-terminal portion of the molecule, which results in ubiquitination and subsequent degradation by the 26S proteasome (Brockman *et al*, 1993). Phosphorylation is mediated by a high molecular weight complex (I-*κ*B kinase or IKK), which in the case of PIF is probably activated by protein kinase C (PKC) (Smith *et al*, 2004). The polyunsaturated fatty acid, eicosapentaenoic acid (EPA), which has been shown to preserve muscle mass in cancer cachexia by downregulating the increased expression of the ubiquitin–proteasome pathway (Whitehouse *et al*, 2001), prevented PIF-induced nuclear migration of NF-*κ*B, loss of I-*κ*B*α* and the increase in proteasome expression (Whitehouse and Tisdale, 2003). This suggests that agents that interfere with activation of NF-*κ*B may be useful in preventing muscle protein degradation in cancer cachexia.

Two potential agents have been studied to verify this hypothesis. Curcumin, a natural product from tumeric, prevents activation of NF-*κ*B by blocking phosphorylation and subsequent degradation of I-*κ*B*α* and has been shown to increase the rate and extent of muscle regeneration after trauma (Thaloor *et al*, 1999). Resveratrol, a natural phytoalexin found in red wine also inhibits NF-*κ*B activation through inhibition of IKK (Holmes-McNary and Baldwin, 2000). Both agents have been evaluated for their ability to inhibit PIF-induced proteasome expression *in vitro* and as anticachectic agents in mice bearing the MAC16 tumour, which induces profound cachexia involving wasting of skeletal muscle (Beck and Tisdale, 1987).

## MATERIALS AND METHODS

### Animals

Pure strain male NMRI mice (average weight 25 g) were obtained from our own inbred colony and were fed a rat and mouse breeding diet (Special Diet Services, Witham, UK) and water *ad libitum*. Animals were implanted with the MAC16 tumour s.c. in the flank by means of a trochar, selecting from donor animals with established weight loss (Beck and Tisdale, 1987). Animals with an average weight loss of 5% (10–12 days after transplantation) were randomised to receive resveratrol (1 mg kg^−1^ body weight dissolved in DMSO : PBS (1 : 20)) i.p. daily, while controls received solvent alone. Body weight and tumour volume were measured daily. Tumour volume was calculated from the formula: Length × (width)^2^/2. All animal experiments followed a strict protocol, approved by British Home Office, and the ethical guidelines that were followed meet the standards required by the UKCCR guidelines (Workman *et al*, 1998). Animals were terminated by cervical dislocation and the soleus muscles were quickly dissected out, together with intact tendons, and maintained in isotonic ice-cold saline before determination of protein degradation. For protein degradation, muscles were fixed by their tendons at approximately resting length in 3 ml of oxygenated (95% oxygen : 5% carbon dioxide) Krebs–Henseleit Buffer (pH 7.4) containing 5 mM glucose and 0.5 mM cycloheximide. The protein degradation rate was determined by the release of tyrosine (Waalkes and Udenfriend, 1957) over a 2 h period.

### Materials

Foetal calf serum (FCS), horse serum (HS) and Dulbecco's modified Eagle's medium (DMEM) were purchased from Life Technologies (Paisley, UK). Mouse monoclonal antibodies to 20S proteasome *α*-subunits, MSS1 and p42 were purchased from Affiniti Research Products (Exeter, UK), while mouse monoclonal antibody to myosin heavy chain was from Novocastra (Newcastle, UK) and to I-*κ*B*α* was from Biomol Research Laboratories Inc. (PA, USA). Rabbit polyclonal antisera to mouse actin was from Sigma Aldridge (Dorset, UK) and to ubiquitin conjugating enzyme (E2_14k_) was a gift from Dr Simon Wing, McGill University, Montreal, Canada. Peroxidase-conjugated rabbit anti-mouse antibody and peroxidase-conjugated goat anti-rabbit antibody were purchased from Dako Ltd. (Cambridge, UK). Hybond A nitrocellulose membranes and enhanced chemiluminescence (ECL) development kits were from Amersham International (Bucks, UK). Electrophoretic-mobility shift (EMSA) gel shift assay kits were from Panomics (California, USA). SN50 and its inactive congener (Lin *et al*, 1995) were purchased from Calbiochem (Nottingham, UK). Curcumin was purchased from Sigma Aldridge (Dorset, UK), and resveratrol from Biomol Research Laboratories Inc. (PA, USA).

### Purification of PIF

Proteolysis-inducing factor was purified from solid MAC16 tumours excised from mice with a weight loss between 20 and 25% as previously described (Todorov *et al*, 1996a; Whitehouse and Tisdale, 2003). Tumours were homogenised in 10 mM Tris-HCl, pH 8.0, containing 0.5 mM phenylmethylsulphonyl fluoride, 0.5 mM EGTA and 1 mM dithiothreitol (DTT) at a concentration of 5 ml g^−1^ tumour. The supernatant obtained after addition of ammonium sulphate (40% w v^−1^) was subjected to affinity chromatography using anti-PIF monoclonal antibody coupled to a solid matrix. The immunogenic fractions were concentrated and used for further studies. The purity of the PIF was confirmed by polyacrylamide gel electrophoresis and immunoblotting. This showed a band for PIF at *M*_r_ 24 000, sometimes accompanied by an albumin-bound band at *M*_r_ 69 000 (Todorov *et al*, 1996b). No other bands were apparent.

### Cell culture

C_2_C_12_ myoblasts were propagated in DMEM supplemented with 10% FCS, glutamine and 1% penicillin–streptomycin under an atmosphere of 10% CO_2_ in air at 37°C. Myotubes were formed by allowing confluent cultures of myoblasts to differentiate in DMEM containing 2% HS with medium changes every 2 days. Differentiation was complete in 5–7 days, and the cells remained viable for a further 4–5 days.

### Measurement of protein degradation

This was determined as described previously (Whitehouse and Tisdale, 2003) by prelabelling cells for 24 h with L-[2,6^3^H]phenylalanine (0.67 mCi mmole^−1^) followed by extensive washing in PBS and further incubation for 2 h in DMEM without phenol red until no more radioactivity appeared in the supernatant. Protein degradation was determined by the release of [2,6^3^H]phenylalanine into the medium after 24 h in the presence of various concentrations of PIF together with 2 mM cold phenylalanine to prevent reincorporation of radioactivity in the cells.

### Measurement of proteasome activity

‘Chymotrypsin-like’ enzyme activity was determined fluorimetrically by the method of Orino *et al* (1991) by the release of aminomethyl coumarin (AMC) from the fluorogenic peptide succinyl-LLVY-AMC. This method has been described previously for C_2_C_12_ myotubes (Whitehouse and Tisdale, 2003). Activity was measured in the absence and presence of the specific proteasome inhibitor lactacystin (10 *μ*M). Only lactacystin suppressible activity was considered to be proteasome specific.

### Western blot analysis

Myotubes were incubated with various concentrations of PIF as depicted in the figure legends, after which the medium was removed and the cells were washed with PBS and scraped from the plastic surface. They were then sonicated at 4°C in 500–2000 *μ*l of 20 mM Tris-HCl, pH 7.5, 2 mM ATP, 5 mM MgCl_2_ and 1 mM DTT. Samples of cytosolic protein (5–30 *μ*g), formed by centrifugation at 18 000 **g** for 5 min, were resolved on 12% sodium dodecylsulphate, polyacrylamide gels (SDS/PAGE) and transferred to 0.45  *μ*m nitrocellulose membranes, which had been blocked with 5% Marvel in Tris-buffered saline, pH 7.5, at 4°C overnight. The primary antibodies were used at a dilution of 1 : 1000 except for actin (1 : 200) and myosin (1 : 100), and the secondary antibodies were also used at a dilution of 1 : 1000. Incubation was for 1 h at room temperature and development was by enhanced chemiluminescence (ECL) (Amersham, United Kingdom). Blots were scanned by a densitometer to quantitate differences.

### Electrophoresis mobility shift assay

DNA-binding proteins were extracted from myotubes according to the method of Andrews and Faller (1991), which utilises hypotonic lysis followed by high salt extraction of nuclei. The EMSA-binding assay was carried out using a Panomics EMSA ‘gel shift’ kit according to the manufacturer's instructions.

### Statistical analysis

Differences in means between groups was determined by one-way ANOVA, followed by Tukey's post-test.

## RESULTS

**Figure 1:**
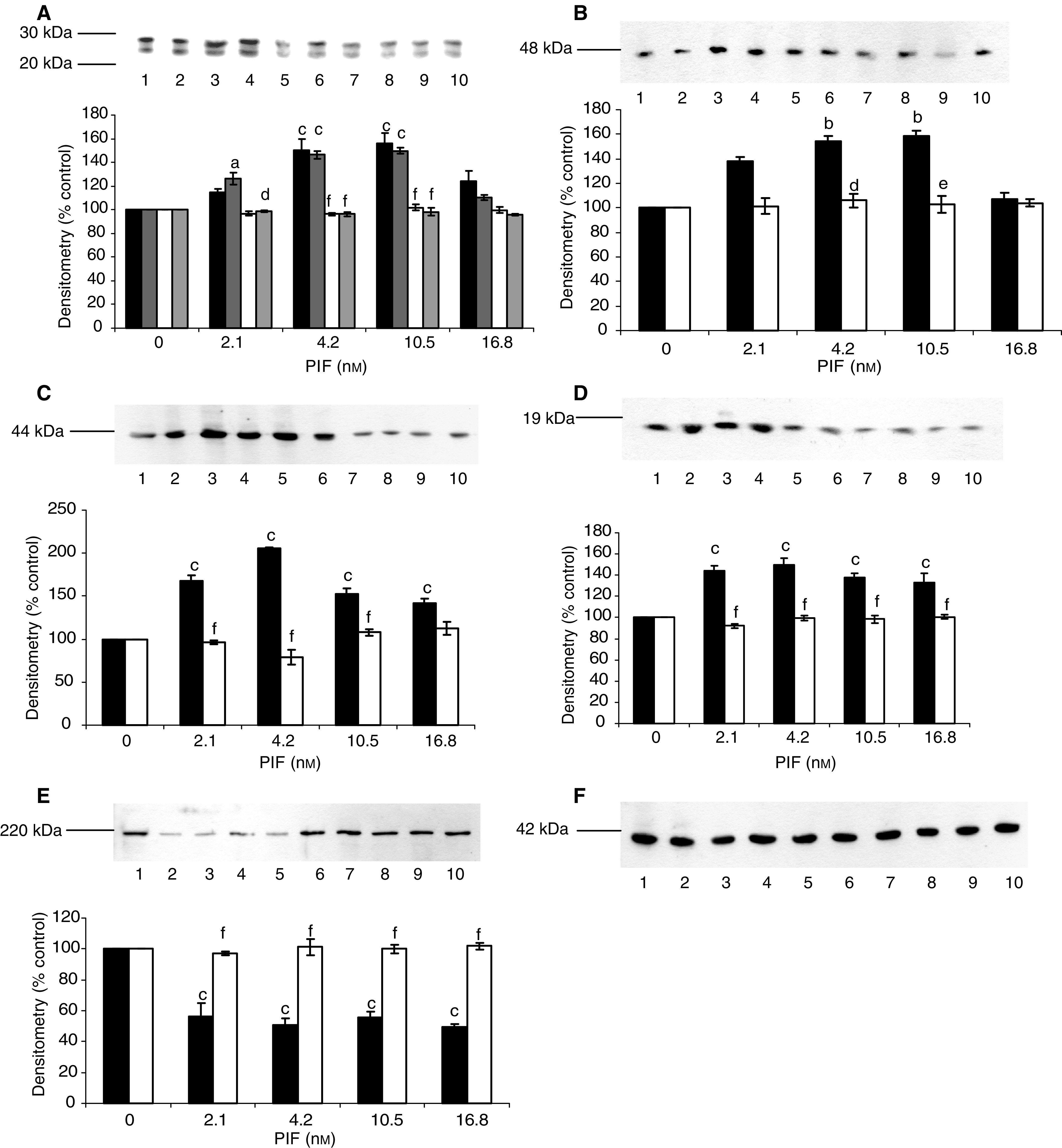
Effect of SN50 (18 *μ*M) (lanes 6–10) and the cell permeable inactive control peptide SN50M (18 *μ*M) (lanes 1–5) on PIF-induced expression of 20S proteasome *α*-subunits (**A**), MSS1 (**B**), p42 (**C**), E2_14k_ (**D**) and myosin (**E**) in murine myotubes 24 h after addition of PIF, as determined by Western blotting. The peptides were added 2 h prior to PIF. An actin loading control is shown in (**F**). Myotubes were treated with 0 (lanes 1 and 6), 2.1 (lanes 2 and 7), 4.2 (lanes 3 and 8), 10.5 (lanes 4 and 9) and 16.8 nM PIF (lanes 5 and 10). A densitometric analysis presenting the average of three separate blots is shown for each Western blot. The densitometric analysis of the proteasome 20S *α*-subunits is for the two major bands in the absence (▪, 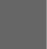) and presence (□, 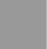) of SN50 (18 *μ*M). Values for the control peptide are shown as solid boxes and for SN50 as open boxes. Differences from control are indicated as a, *P*<0.05, b, *P*<0.01 and c, *P*<0.001, while differences from the control peptide in the presence of SN50 are shown as d, *P*<0.05, e, *P*<0.01 and f, *P*<0.001.

To investigate the importance of activation of NF-*κ*B in PIF-induced proteasome expression and degradation of myofibrillar proteins in murine myotubes, the cell permeable specific NF-*κ*B inhibitor SN50 (18 *μ*M) was used to block agonist-induced nuclear translocation of NF-*κ*B (Lin *et al*, 1995). At this concentration, SN50 is not cytotoxic, and there have been no reports that it interferes with any other signalling pathway. The PIF-induced increase in 20S proteasome *α*-subunits seen in the presence of the inactive congener was not seen in the presence of SN50 (Figure 1A

). Similar results were seen for PIF-induced stimulation of MSS1, an ATPase subunit of the 19S regulatory complex (Figure 1B), p42, an ATPase subunit of the 19S regulator that promotes ATP-dependent association of the 20S proteasome with the 19S regulator to form the 26S proteasome (Tanahashi *et al*, 1999) (Figure 1C), and E2_14k_, one of the major mammalian E2s that support E3*α*-dependent ubiquitin conjugate formation (Attaix *et al*, 1999) (Figure 1D), as well as the PIF-induced decrease in myosin expression (Figure 1E). In each case, PIF produced an effect between 2.1 and 10.5 nM, which was not seen in the presence of SN50. These results suggest that nuclear translocation of NF-*κ*B is essential for PIF-induced proteasome expression, ubiquitin conjugation and loss of the myofibrillar protein myosin.

**Figure 2:**
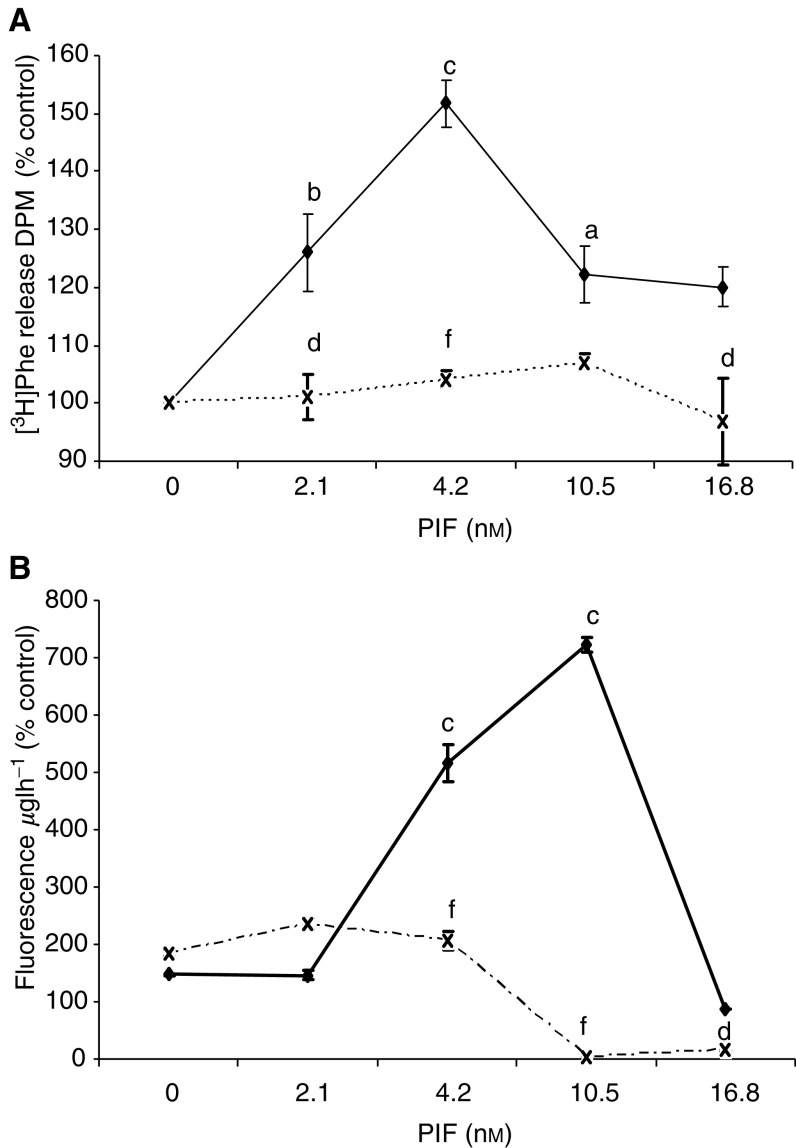
(**A**) Effect of curcumin (50 *μ*M) on total protein degradation in the presence of PIF for 24 h, as measured by the release of [^3^H]phenylalanine. Myotubes were incubated with PIF alone (⧫) or pretreated with curcumin (×) 2 h prior to the addition of PIF. Differences from 0 nM PIF are indicated as b, *P*<0.01 and c, *P*<0.001, while differences in the presence of curcumin are indicated as d, *P*<0.05 and f, *P*<0.001. *n*=9. The experiment was repeated three times. (**B**) Chymotrypsin-like enzyme activity in soluble extracts of murine myotubes after treatment with PIF alone for 24 h (⧫), or after treatment with PIF in the presence of curcumin (×). The experiment was repeated three times (*n*=9). The symbols for the differences are the same as in (**A**).

**Figure 3:**
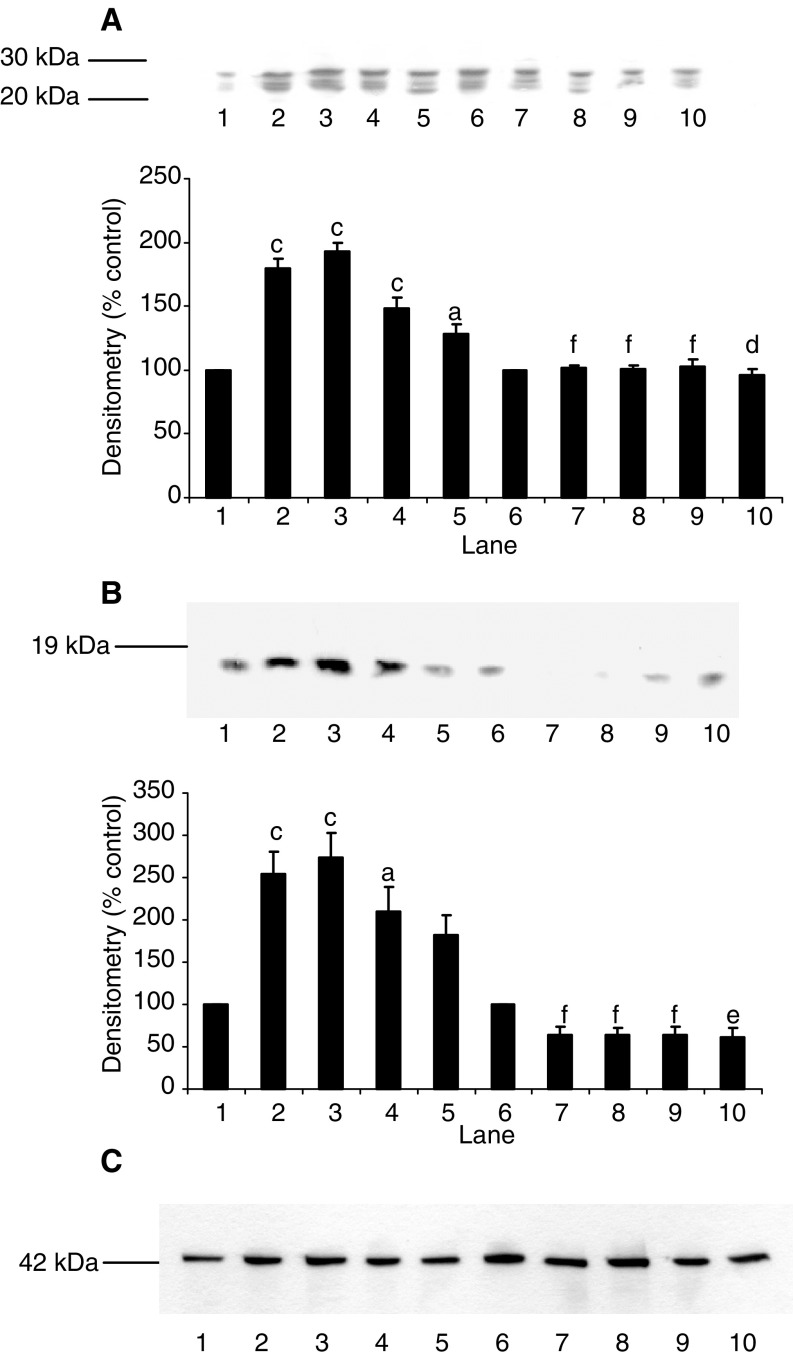
Effect of curcumin on PIF-induced expression of 20S proteasome *α*-subunits (**A**) and E2_14k_ (**B**) in murine myotubes, determined by Western blotting. Cells were incubated for 24 h with 0 (lanes 1 and 6), 2.1 (lanes 2 and 7), 4.2 (lanes 3 and 8), 10.5 (lanes 4 and 9) or 16.8 nM PIF (lanes 5 and 10) in the absence (lanes 1–5) or presence (lanes 6–10) of curcumin (50 *μ*M) added 2 h prior to PIF. An actin loading control is shown in (**C**). Densitometric analysis of three separate blots is shown under each Western blot. Differences from 0 nM PIF are shown as a, *P*<0.05, b, *P*<0.01 and c, *P*<0.001, while differences in the presence of curcumin are indicated as d, *P*<0.05, e, *P*<0.01 and f, *P*<0.001.

**Figure 4:**
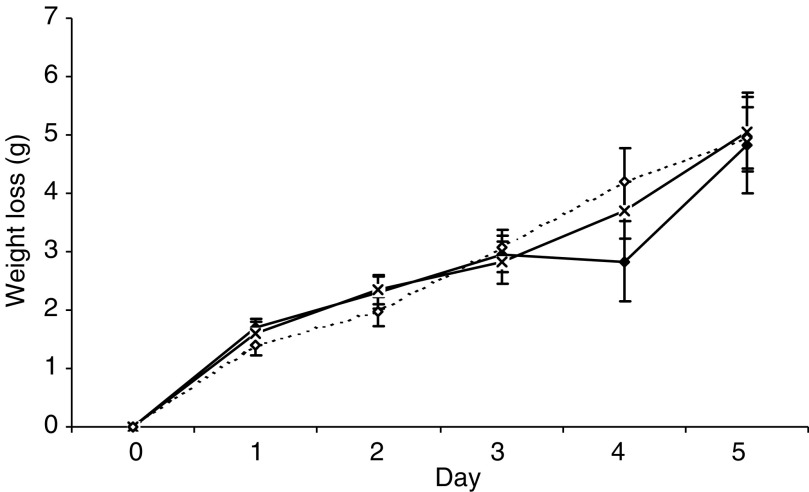
Effect of curcumin administered p.o. daily at 150 (×) or 300 (◊) mg kg^−1^ in DMSO : PBS (1 : 1000), as previously used (Busquets *et al*, 2001), compared with DMSO : PBS (1 : 1000) alone (⧫) on weight loss in mice bearing the MAC16 tumour over a 5 day period. There was a significant weight loss for all groups at day 5 (*P*<0.001), but there was no difference in weight loss between groups.

To evaluate the effect of pharmacological inhibition of NF-*κ*B on PIF-induced proteasome expression and protein degradation, the IKK inhibitor curcumin (Thaloor *et al*, 1999) was employed. At a concentration of 50 *μ*M, curcumin completely attenuated total protein degradation in murine myotubes at all concentrations of PIF (Figure 2A

). In addition, curcumin attenuated the PIF-induced increase in ‘chymotrypsin-like’ enzyme activity (Figure 2B), the predominant proteolytic activity of the proteasome, as well as the increase in 20S proteasome *α*-subunits and E2_14k_ (Figure 3

). These results suggest that curcumin may be effective in the treatment of muscle atrophy in cancer cachexia. However, when curcumin was evaluated *in vivo* in mice bearing the MAC16 tumour, it was shown to be ineffective in preventing loss of body weight at dose levels of 150 and 300 mg kg^−1^ (Figure 4

). These results prompted us to look for alternative inhibitors of NF-*κ*B, which may be effective *in vivo*.

**Figure 5:**
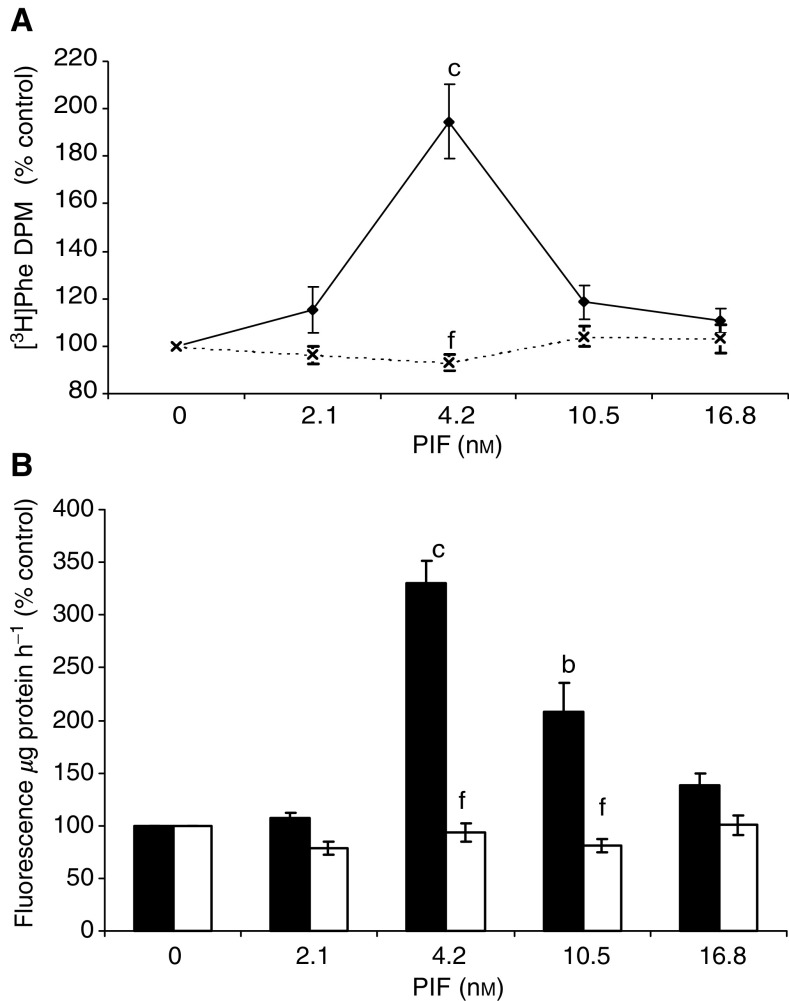
(**A**) Effect of resveratrol (30 *μ*M) on total protein degradation in murine myotubes in the presence of PIF for 24 h, as measured by the release of [^3^H]phenylalanine. Myotubes were incubated with PIF alone (⧫) or preincubated with resveratrol 2 h prior to addition of PIF and maintained in the culture medium over the 24 h period (×). Differences from control are indicated as b, *P*<0.01 and c, *P*<0.001, while differences in the presence of resveratrol are indicated as f, *P*<0.001. The experiment was repeated three times (*n*=9). (**B**) Chymotrypsin-like enzyme activity in soluble extracts of murine myotubes after treatment with PIF alone for 24 h (solid boxes) or after treatment with PIF in the presence of resveratrol (30 *μ*M) (open boxes). The symbols for the differences are the same as in (**A**). The experiment was repeated three times (*n*=9).

**Figure 6:**
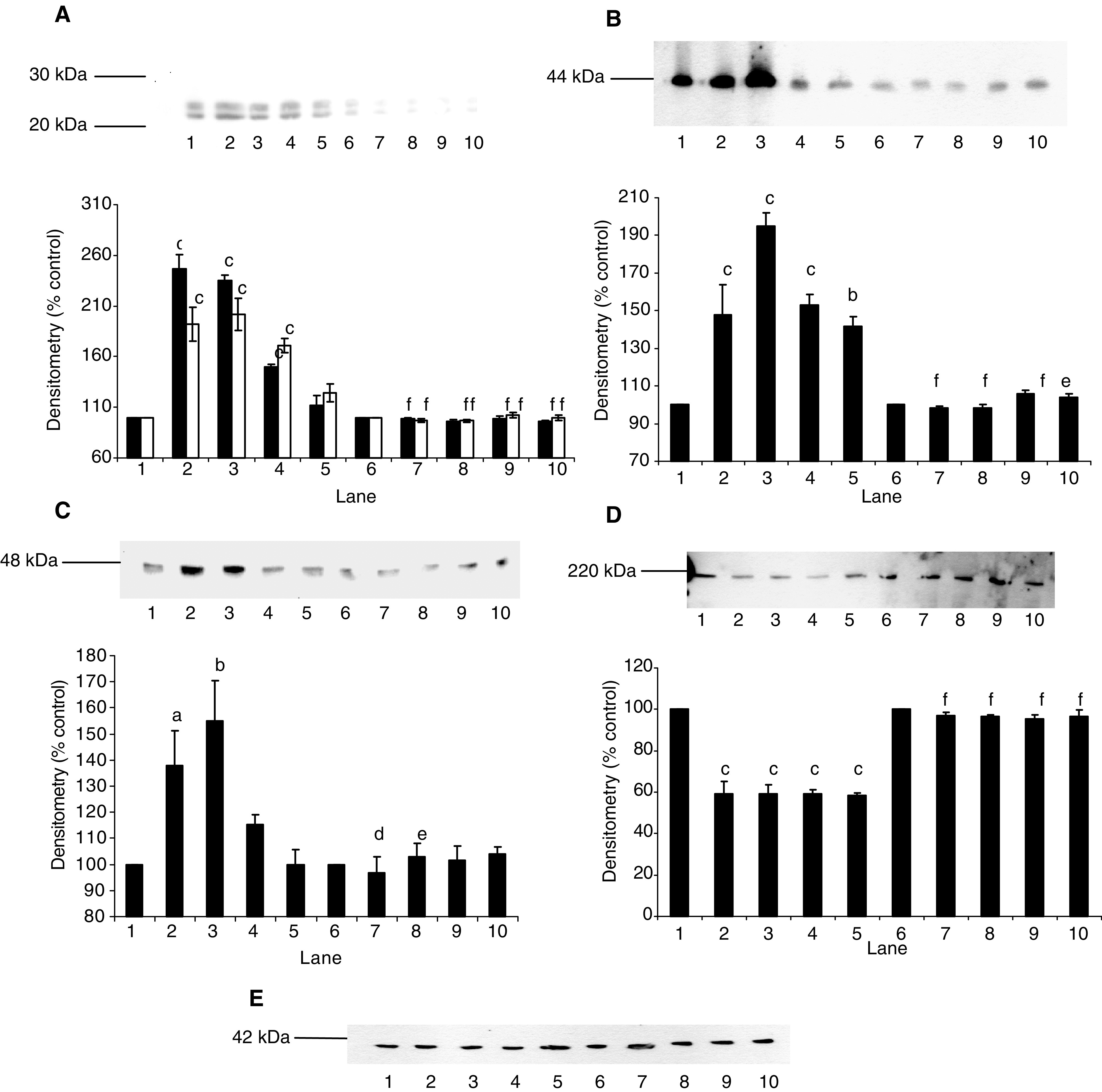
Effect of resveratrol on PIF-induced expression of 20S proteasome *α*-subunits (**A**), p42 (**B**), MSS1 (**C**) and myosin (**D**) as determined by Western blotting. An actin loading control is shown in (**E**). Cells were incubated with 0 (lanes 1 and 6), 2.1 (lanes 2 and 7), 4.2 (lanes 3 and 8), 10.5 (lanes 4 and 9) or 16.8 nM PIF (lanes 5 and 10) in the absence (lanes 1–5) or presence (lanes 6–10) of resveratrol (30 *μ*M) for 24 h. A densitometric analysis of the three separate Western blots is shown underneath a representative example. Differences from control are indicated as a, *P*<0.05, b, *P*<0.01 and c, *P*<0.001, while differences in the presence of resveratrol are indicated as d, *P*<0.05, e, *P*<0.01 and f, *P*<0.001.

**Figure 7:**
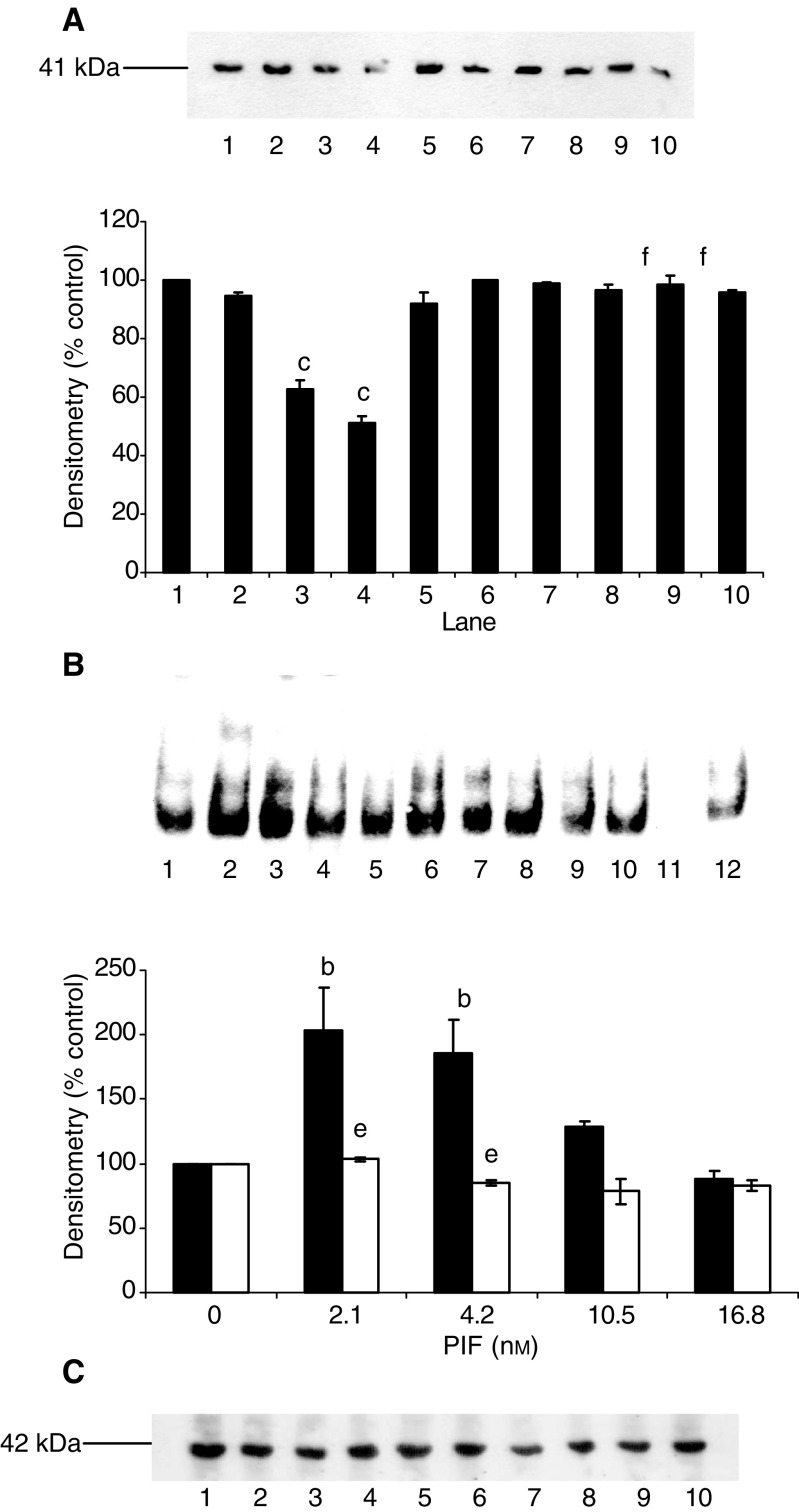
Effect of PIF on I-*κ*B*α* expression (**A**) and nuclear translocation of NF-*κ*B, as determined by EMSA (**B**) in murine myotubes 30 min after the addition of PIF. An actin loading control for (**A**) is shown in (**C**). Myotubes were incubated with 0 (lanes 1 and 6), 2.1 (lanes 2 and 7), 4.2 (lanes 3 and 8), 10.5 (lanes 4 and 9) or 16.8 nM PIF (lanes 5 and 10) in the absence (lanes 1–5) or presence (lanes 6–10) of resveratrol (30 *μ*M). In (**B**), lane 12 is a positive control for NF-*κ*B (supplied by the manufacturer of the kit), while lane 11 contains the positive control for NF-*κ*B together with a 100-fold excess of unlabelled NF-*κ*B probe. The densitometric analysis represents three separate experiments. Differences from control are indicated as b, *P*<0.01 and c, *P*<0.001, while differences in the presence of resveratrol are indicated as e, *P*<0.01 and f, *P*<0.001.

Thus, the effect of another inhibitor of IKK, resveratrol (Holmes-McNary and Baldwin, 2000) on PIF-induced protein degradation and proteasome expression was studied in murine myotubes. At a concentration of 30 *μ*M, resveratrol effectively attenuated both PIF-induced protein degradation (Figure 5A

), as well as the proteasome chymotrypsin-like enzyme activity (Figure 5B). Resveratrol also attenuated the PIF-induced increase in 20S proteasome *α*-subunit expression (Figure 6A

), p42 (Figure 6B), MSS1 (Figure 6C), as well as the decrease in myosin expression produced by PIF (Figure 6D). To confirm that resveratrol produced effects on NF-*κ*B at this concentration, murine myotubes were treated with resveratrol (30 *μ*M) 2 h prior to the addition of PIF and the effect on I-*κ*B*α* degradation and nuclear accumulation of NF-*κ*B was determined (Figure 7

). Both PIF-induced degradation of I-*κ*B*α* (Figure 7A), and nuclear binding of NF-*κ*B in response to PIF (Figure 7B) were effectively inhibited by resveratrol, confirming that the mechanism of action in murine myotubes was as expected.

**Figure 8:**
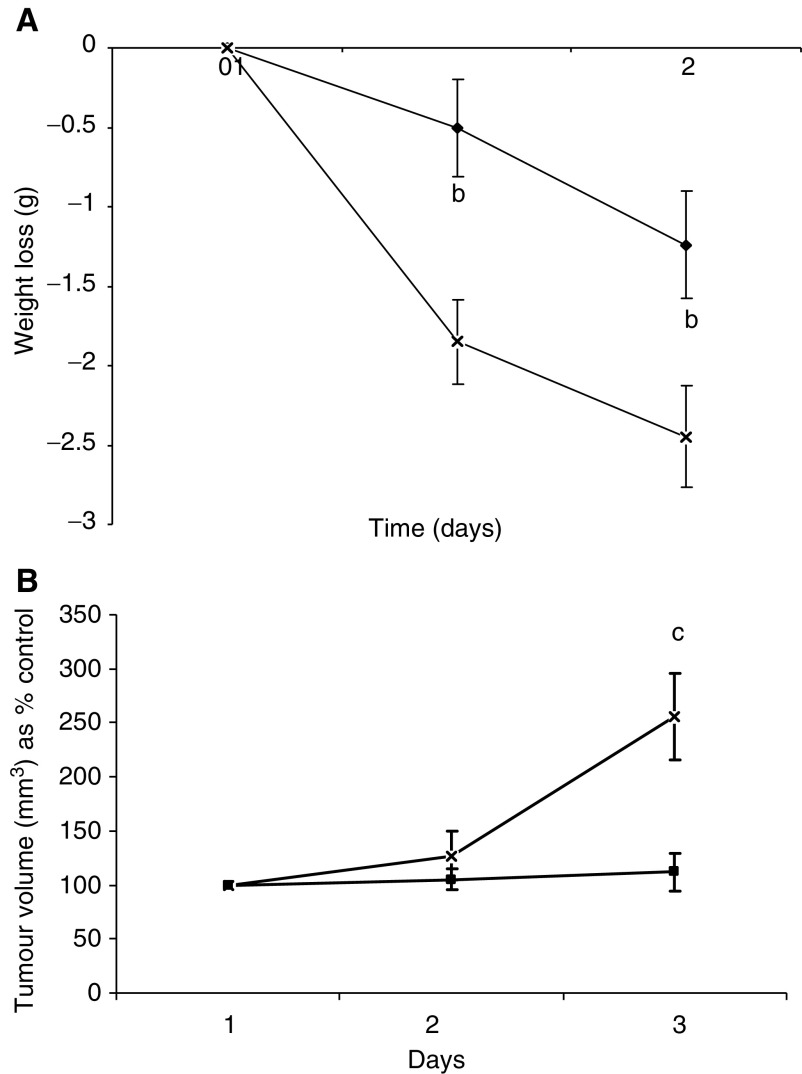
Effect of resveratrol (1 mg kg^−1^ ⧫) on body weight (**A**) and tumour volume (**B**) in mice bearing the MAC16 colon adenocarcinoma compared with solvent (DMSO : PBS; 1 : 20) controls (×). The number of mice in each group, *n*=6. Differences from control are indicated as b, *P*<0.01 and c, *P*<0.001.

**Figure 9:**
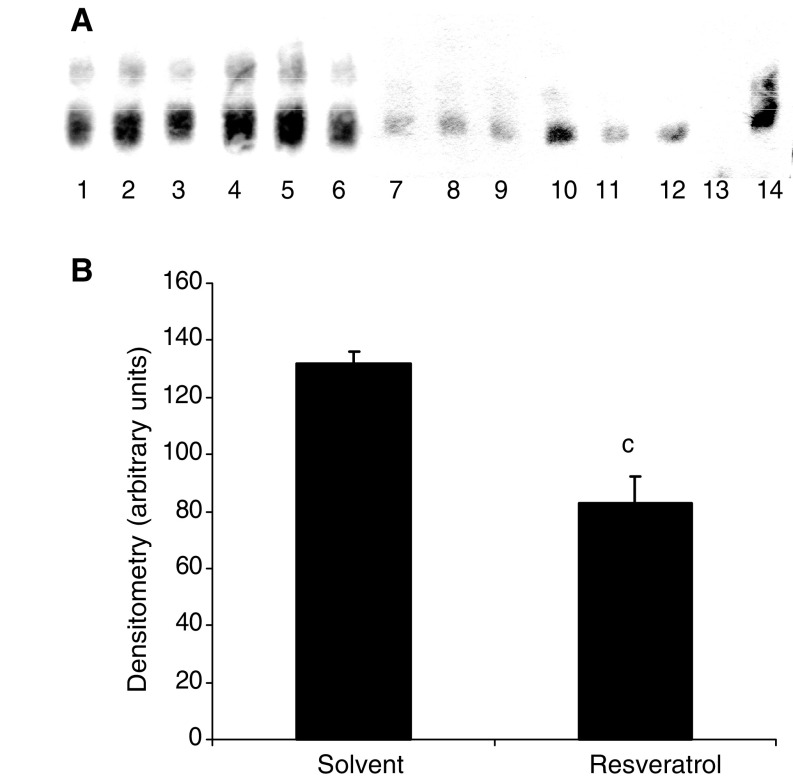
(**A**) EMSA of nuclear binding of NF-*κ*B in gastrocnemius muscle of mice receiving solvent control (lanes 1–6) or resveratrol (1 mg kg^−1^) (lanes 7–12) after 48 h treatment. Lane 14 is the positive control for NF-*κ*B and lane 13 is the positive control in the presence of a 100-fold excess of unlabelled NF-*κ*B probe. (**B**) Densitometric analysis of the EMSA shown in (**A**), *n*=3. Differences from control are shown as c, *P*<0.001.

To evaluate the anticachectic effect of resveratrol, mice bearing the MAC16 tumour, and with established weight loss (5%) were treated with resveratrol (1 mg kg^−1^) daily and the effect on body weight and tumour volume were determined (Figure 8

). Resveratrol significantly attenuated weight loss within 24 h of administration (Figure 8A) and this was accompanied by inhibition of tumour growth within 48 h (Figure 6B). In order to determine whether the increase in body weight in mice treated with resveratrol resulted from a reduction in protein degradation, the rate of protein degradation in soleus muscle was determined by the release of tyrosine (Figure 9

). Previous studies (Beck *et al*, 1991) have shown that muscles from mice bearing the MAC16 tumour have a significant increase in protein degradation compared with nontumour-bearing controls. Soleus muscle of mice bearing the MAC16 tumour and treated with resveratrol had a significant reduction in protein degradation after 48 h treatment compared to muscles from mice treated with solvent alone (from 955±125 to 568±4.5 fluorescent units g^−1^ 2 h^−1^, *P*<0.001, *n*=12). To verify that this arose from an effect on nuclear migration of NF-*κ*B, the amount of NF-*κ*B in the nucleus was determined by EMSA. The results depicted in Figure 9 show a significant reduction in NF-*κ*B DNA-binding activity in gastrocnemius muscles of mice treated with resveratrol. These results suggest that agents which inhibit nuclear translocation of NF-*κ*B may prove useful in the treatment of muscle wasting in cancer cachexia.

## DISCUSSION

Nuclear factor-*κ*B is involved in the control of a large number of cellular processes, such as immune and inflammatory responses, developmental processes, cell growth and apoptosis (Baldwin, 1996). Activation of NF-*κ*B has been connected with tumour cell survival and proliferation, invasion and angiogenesis, critical events in tumour metastasis, as well as resistance to chemotherapy (Baldwin, 2001). Nuclear factor-*κ*B has also been shown to mediate the protein loss induced by tumour necrosis factor-*α* (TNF-*α*) in differentiated skeletal muscle myotubes (Li and Reid, 2000), and to be an important transcription factor in PIF-induced proteasome expression in murine myotubes (Whitehouse and Tisdale, 2003). Nuclear factor-*κ*B may also be involved in other conditions of muscle wasting. Thus, hind-limb unloading, a model of disuse atrophy, resulted in a 10-fold increase in the activity of a transfected NF-*κ*B-dependent reporter (Hunter *et al*, 2002), but this involved p50, c-Rel and Bcl-3 and not activation of p65 or I-*κ*B, and is thus distinct from cachexia.

This suggests that inhibitors of the release and subsequent nuclear translocation of NF-*κ*B, or the binding of NF-*κ*B to DNA should be evaluated as potential agents to prevent muscle atrophy in cancer cachexia. Agonist-induced nuclear translocation of NF-*κ*B in intact cells can be inhibited by cell-permeable synthetic peptides representing the nuclear localisation sequence of NF-*κ*B (Lin *et al*, 1995). When such a peptide (SN50) was incubated together with PIF in murine myotubes it attenuated both the PIF-induced proteasome expression and the ubiquitin conjugating enzyme, E2_14k_, as well as the loss of myofibrillar protein myosin. These results suggest that inhibition of NF-*κ*B activation should inhibit muscle protein degradation induced by PIF in cancer cachexia.

Both curcumin and resveratrol, known to be inhibitors of IKK, attenuated PIF-induced protein degradation in murine myotubes, as well as the increased expression of the ubiquitin–proteasome proteolytic pathway. Systemic administration of curcumin (20 *μ*g kg^−1^) has previously been shown not to influence muscle wasting or changes in body weight in rats bearing the highly cachectic Yoshida AH-130 ascites hepatoma, although it did reduce tumour growth (Busquets *et al*, 2001). In the present study, curcumin was also unable to reduce weight loss in mice bearing the MAC16 tumour, even at much higher doses (150 and 300 mg kg^−1^). This may be due to the low bioavailability (0.06% absorption) (Pan *et al*, 1999), which is probably insufficient to exert a therapeutic effect. Even a dose of 1 g kg^−1^ body weight in the mouse yielded a plasma level of only 0.5 *μ*M (Pan *et al*, 1999), which is below the concentration needed to attenuate PIF action *in vitro*. Resveratrol (1 mg kg^−1^) was also unable to prevent the loss of body weight in rats bearing the Yoshida hepatoma, although again it did reduce tumour growth rate (Carbó *et al*, 1999). In this study, resveratrol at the same concentration (1 mg kg^−1^) was found to attenuate weight loss, followed by a reduction in tumour growth rate in mice bearing the MAC16 tumour, reduce protein degradation in skeletal muscle, as determined by the release of tyrosine, and reduce nuclear translocation of NF-*κ*B in gastrocnemius muscle. The reason for the difference in response to resveratrol in the two models is not clear. Although TNF-*α* has been implicated in the enhanced protein degradation in muscle of rats bearing the Yoshida ascites hepatoma (Costelli *et al*, 1993), while in mice bearing the MAC16 carcinoma PIF appears to be responsible for the enhanced protein degradation (Lorite *et al*, 1998), NF-*κ*B has been shown to mediate the protein loss induced by TNF-*α* (Li and Reid, 2000) and thus inhibitors of NF-*κ*B should also be effective in this model.

Other inhibitors of NF-*κ*B have established or potential roles in the treatment of cachexia. Thus EPA, which prevents nuclear migration of NF-*κ*B in murine myotubes in response to PIF by stabilising the cytosolic I-*κ*B/NF-*κ*B complex (Whitehouse and Tisdale, 2003), possibly by interfering with I-*κ*B phosphorylation (Novak *et al*, 2003), has been shown to attenuate the development of further weight loss in weight-losing patients with pancreatic cancer (Wigmore *et al*, 2000) and produce an increase in lean body mass when combined with a high protein energy dense nutritional supplement (Barber *et al*, 1999). The anti-inflammatory agent, ibuprofen, has been shown to inhibit constitutive activation of NF-*κ*B and IKK*α* in prostate cancer cells (Palayoor *et al*, 1999), and when combined with megestrol acetate produced an increase in body weight in gastrointestinal cancer patients (McMillan *et al*, 1999), while megestrol acetate alone produced a decrease in body weight. Another anti-inflammatory agent, thalidomide, can also block NF-*κ*B activation through inhibition of IKK (Keifer *et al*, 2001), and has been shown to promote weight gain in HIV-infected patients receiving treatment for tuberculosis (Reys-Teran *et al*, 1996), and is currently under evaluation for the treatment of cancer cachexia. These results suggest that despite the fact that NF-*κ*B is involved in the control of over 150 target genes (Pahl, 1999), inhibitors of NF-*κ*B activation do not produce overt toxicity and should be evaluated for the treatment of muscle atrophy in cancer cachexia.
